# Supplementary material for: Cortical oscillatory dysrhythmias in visual snow syndrome: a magnetoencephalography study
Source: Brain Commun. 2021 Dec 18;4(1):fcab296. doi: 10.1093/braincomms/fcab296 (PMC8833316; doi:10.1093/braincomms/fcab296)
Supplement: fcab296_Supplementary_Data [file fcab296_supplementary_data.pdf]

Supplementary Materials

Cortical Oscillatory Dysrhythmias in Visual Snow Syndrome: a magnetoencephalography study

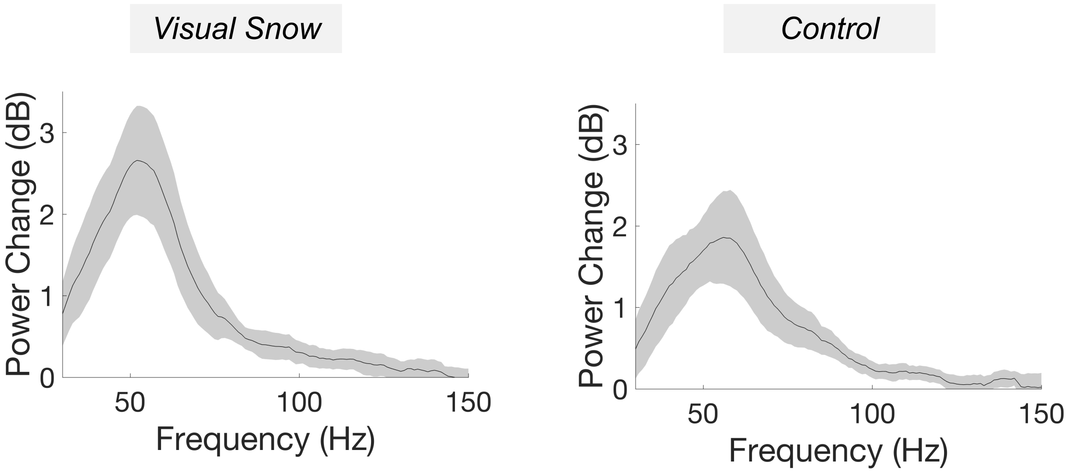

Supplementary Figure 1: Gamma-band power change in the V1 region of interest, following presentation of the visual grating. The solid line represents the groups mean. Shaded error bars correspond to 95% confidence intervals. Note, the peak in the frequency spectrum from 40-70Hz across both groups.

Visual Snow

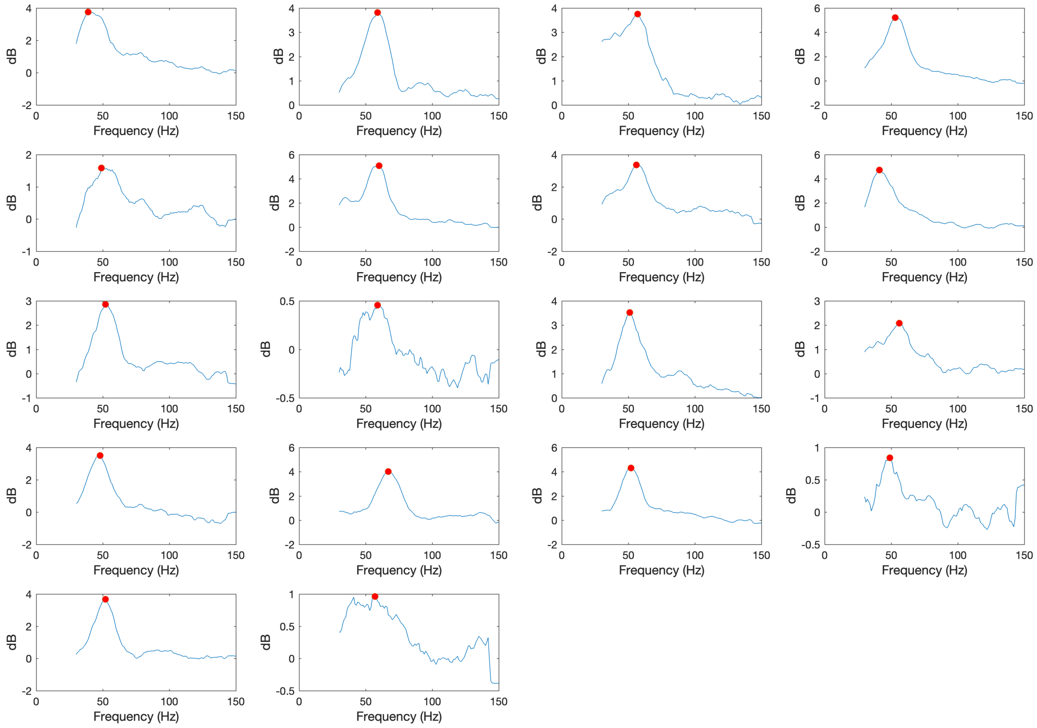

Supplementary Figure 2a: For each VSS participant, the change in gamma-band power following grating presentation in V1 is plotted, alongside the results of the peak-finding (using *findpeaks.m*)

Control

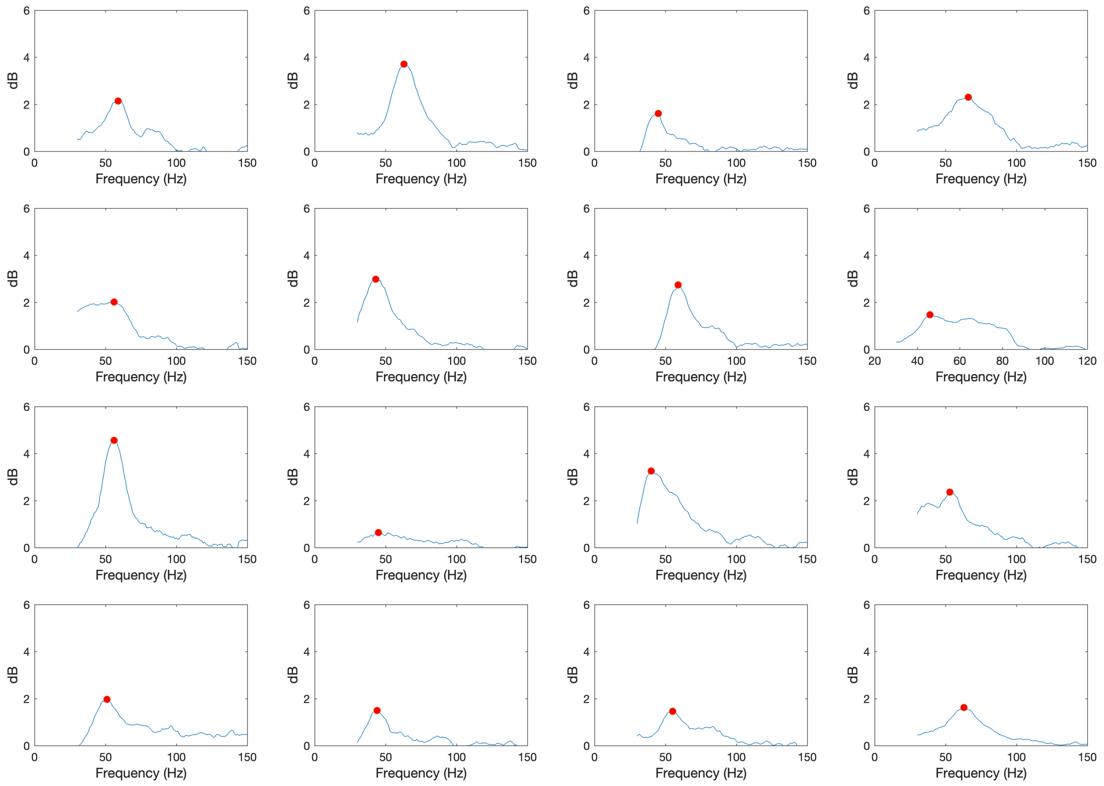

Supplementary Figure 2b: For each control participant, the change in gamma-band power following grating presentation in V1 is plotted, alongside the results of the peak-finding (using *findpeaks.m*)

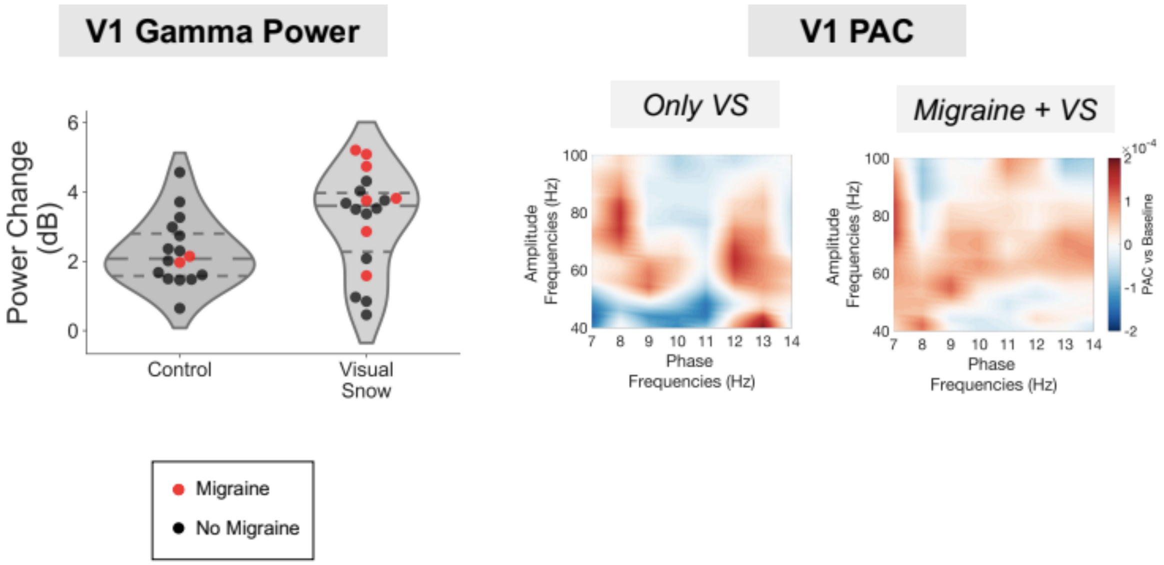

Supplementary Figure 3: V1 gamma power (left) was plotted separately for individual participants with concurrent migraine symptoms (red circles) and without (black dots). Similarly, group-level V1 alpha-gamma PAC was plotted (right) for visual snow patients with and without concurrent migraine symptoms. In both cases, no clear pattern can be observed between the two sub-groups.

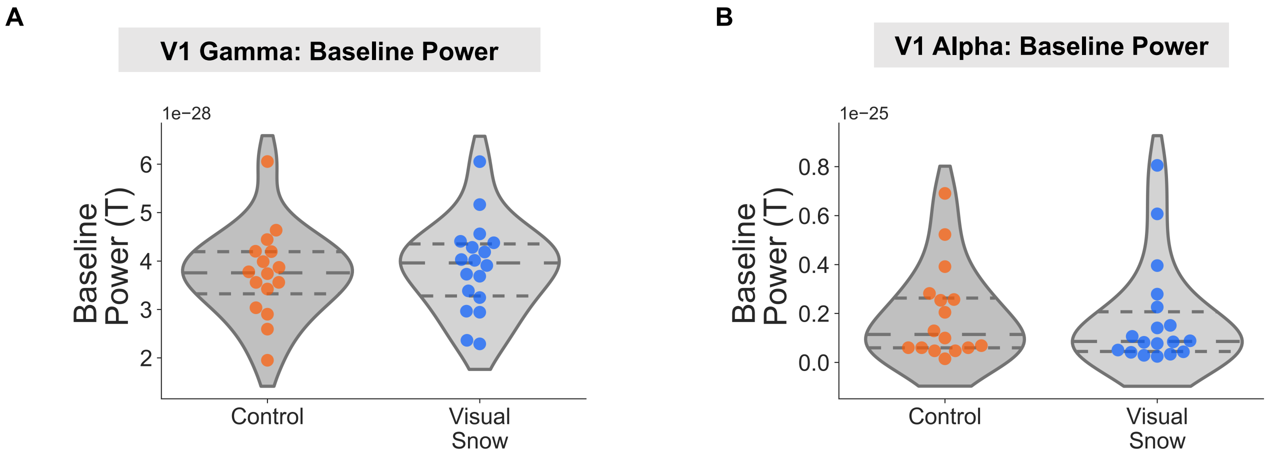

Supplementary Figure 4: Data from the V1 region of interest was examined for potential group differences in baseline time-period (-1.5 to -0.2s relative to stimulus onset). No statistical differences in baseline power were observed between groups for (A) gamma or (B) alpha power, $p>0.05$. Dots represent individual participants. Violin plots have the median and interquartile range shown with dotted lines. *Group differences were analysed using an independent samples t-test, two-tailed*.

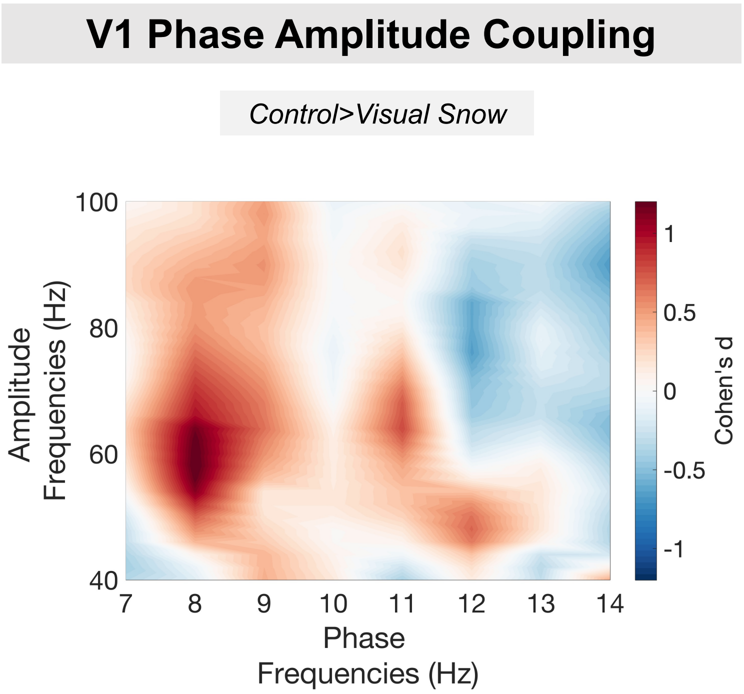

Supplementary Figure 5: The effect size of the V1 PAC group difference was quantified using Cohen's d (`ft_statfun_cohensd`). More details on the specific computational steps can be read here:
https://www.fieldtriptoolbox.org/example/effectsize/

Visual Snow

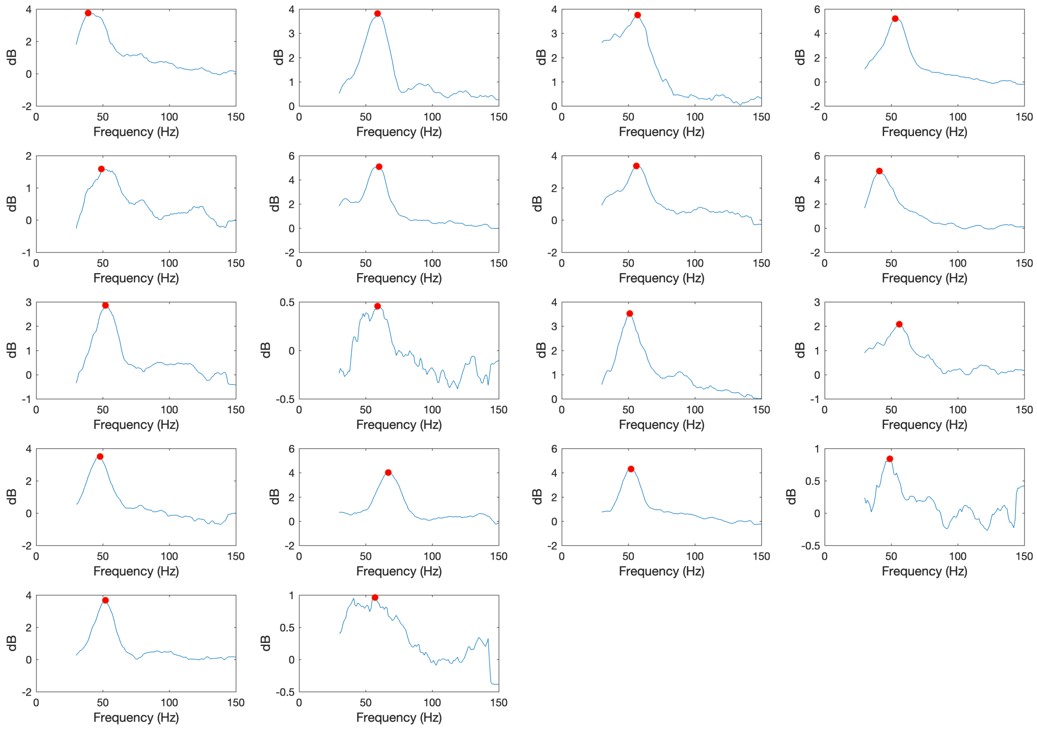

Supplementary Figure (previously 2a): For each VSS participant, the change in gamma-band power following grating presentation in V1 is plotted, alongside the results of the peak-finding (using *findpeaks.m*)

Control

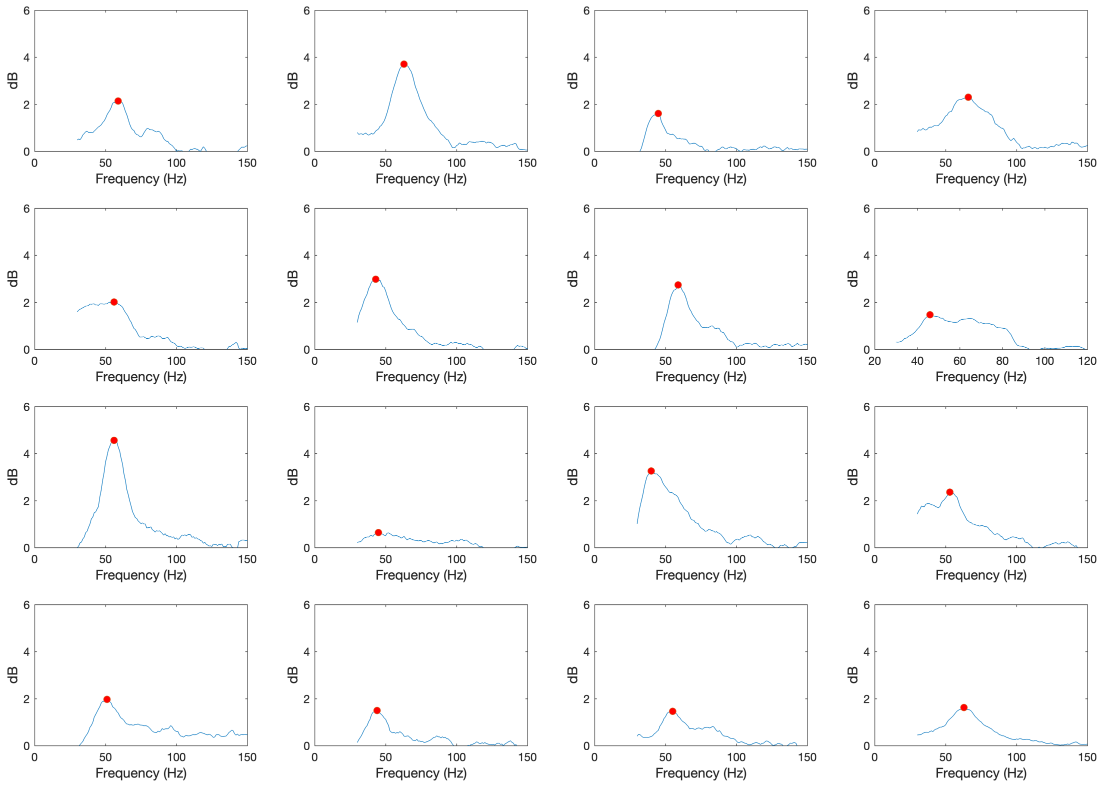

Supplementary Figure (previously 2b): For each control participant, the change in gamma-band power following grating presentation in V1 is plotted, alongside the results of the peak-finding (using *findpeaks.m*)
